# A Robust PDR/UWB Integrated Indoor Localization Approach for Pedestrians in Harsh Environments

**DOI:** 10.3390/s20010193

**Published:** 2019-12-29

**Authors:** Haibin Tong, Ning Xin, Xianli Su, Tengfeng Chen, Jingjing Wu

**Affiliations:** 1 School of Computer Science and Engineering, Northeastern University, Shenyang 110819, China; tong6445@126.com (H.T.); xianlis@126.com (X.S.); wujingjing@cse.neu.edu.cn (J.W.); 2 China Academy of Space Technology, Institute of Telecommunication Satellite, Beijing 100094, China; xin253@hotmail.com

**Keywords:** wireless sensor networks (WSNs), indoor localization, pedestrian dead reckoning (PDR), ultra-wide band (UWB), harsh environments

## Abstract

Wireless sensor networks (WSNs) and the Internet of Things (IoT) have been widely used in industrial, construction, and other fields. In recent years, demands for pedestrian localization have been increasing rapidly. In most cases, these applications work in harsh indoor environments, which have posed many challenges in achieving high-precision localization. Ultra-wide band (UWB)-based localization systems and pedestrian dead reckoning (PDR) algorithms are popular. However, both have their own advantages and disadvantages, and both exhibit a poor performance in harsh environments. UWB-based localization algorithms can be seriously interfered by non-line-of-sight (NLoS) propagation, and PDR algorithms display a cumulative error. For ensuring the accuracy of indoor localization in harsh environments, a hybrid localization approach is proposed in this paper. Firstly, UWB signals cannot penetrate obstacles in most cases, and traditional algorithms for improving the accuracy by NLoS identification and mitigation cannot work in this situation. Therefore, in this study, we focus on integrating a PDR and UWB-based localization algorithm according to the UWB communication status. Secondly, we propose an adaptive PDR algorithm. UWB technology can provide high-precision location results in line-of-sight (LoS) propagation. Based on these, we can train the parameters of the PDR algorithm for every pedestrian, to improve the accuracy. Finally, we implement this hybrid localization approach in a hardware platform and experiment with it in an environment similar to industry or construction. The experimental results show a better accuracy than traditional UWB and PDR approaches in harsh environments.

## 1. Introduction

With the development of wireless sensor networks (WSNs) and the Internet of Things (IoT) in industrial, construction, and other fields, accurate indoor localization technology for pedestrians is playing an increasingly important role. For example, the localization of workers in industry can prevent them from entering dangerous areas. The environments in these fields have their own characteristics. On the one hand, they are all in indoor environments, so satellite-based outdoor localization systems, such as the Global Positioning System (GPS) and BeiDou Navigation Satellite System (BDS), cannot be used due to the weak penetration of the satellite signal. On the other hand, they are harsh environments in most cases. There are some commercial indoor localization systems and mature research prototypes for line-of-sight (LoS) propagation environments, but the accuracy of these solutions is not satisfactory [1,2] in NLoS propagation. However, non-line-of-sight (NLoS) is the most important feature of harsh environments. Indoor localization technology is still very challenging under NLoS propagation in harsh environments. 

The current mainstream indoor localization systems are radio frequency (RF)-based. This kind of system basically requires beacon nodes, or beacon nodes (BNs), with known coordinates in the localization system. Each target to be located has an electronic tag, or tag node (TN). Specific features generated from the communication between the TN and BN are used for localization. These features mainly include the radio signal strength (RSS), time, signal angle, etc. The challenge of indoor localization systems in harsh environments is that these features can be severely disturbed [1] by NLoS propagation or other interference. As a result, the localization accuracy will be greatly reduced.

In harsh environments, interference of an RF-based localization system can be mainly divided into three types. Firstly, multipath refraction is a common type of interference in harsh environments. It means that an RF signal will be transmitted to the receiver through multiple paths, which causes the transmission time to be unreliable and unpredictable RSS superposition. Additionally, it apparently has a greater impact on the signal angle. Secondly, non-line-of-sight (NLoS) propagation caused by obstacles is also a very serious form of interference. It can cause signal attenuation, time delay, and signal interruption. Moreover, it interferes with the identification of multipath by some systems. Thirdly, electromagnetic fields of some large devices can interfere with signal transmission.

An ultra-wide band (UWB)-based localization system, such as an RF-based algorithm, is popular. Since UWB communication has a mature solution for multipath and the ability to record time features, the UWB-based localization algorithm is the most accurate indoor localization solution. Most studies have tried to improve the accuracy of UWB-based localization in harsh environments by NLoS identification and mitigation. However, UWB works in a high frequency, and UWB signals cannot penetrate obstacles in most cases. This makes NLoS identification and mitigation pointless. RF-based indoor localization algorithms have problems achieving high-precision locations in harsh environments, in which cases some hybrid systems have been proposed. This refers to the addition of other auxiliary sensors in the localization system, such as ultrasonic, infrared ranging, and inertial measurement unit (IMU) sensors, etc. The IMU-based pedestrian dead reckoning (PDR) algorithm combined with RF localization is a potential solution in terms of cost and implementation difficulty. However, its related research in harsh environments is still rare.

To address the problem presented above, this paper proposes a hybrid indoor localization system based on integrating PDR/UWB to achieve a high precision in harsh environments for pedestrians. The core idea includes two aspects. First, the UWB-based localization algorithm has a high accuracy in the case of less interference, and based on this, we can obtain the speed and heading of every pedestrian. Second, using the speed and heading, we propose a strategy to dynamically adjust key parameters for each pedestrian in the PDR algorithm. Third, the PDR algorithm is not sensitive to environmental interference, and this advantage can correct location errors caused by environmental interference in the UWB-based algorithm. The contributions of this paper can be summarized as follows:

(1) In localization environments with LoS, the UWB-based algorithm can provide a high-precision trajectory for each individual. We propose a strategy to identify this localization situation, and using the location results, we estimate the speed and heading for each pedestrian;
(2) An improved PDR algorithm is proposed, which can dynamically adjust the parameters of each pedestrian’s stride estimation and heading prediction; and
(3) The final location results will be calculated using a weighted average algorithm, wherein the weights are dynamically adjusted according to the UWB communication condition.

The rest of this paper is organized as follows: Section 2 gives a short summary of the related works for indoor localization in harsh environments. Section 3 summarizes the problem of localization in harsh environments and briefly describes the system architecture. Then, the UWB-based and PDR localization algorithm is proposed in Section 4 and Section 5. Section 6 provides the fusion of results from two localization algorithms. Section 7 and Section 8 detail the experimental results and conclusions. 

## 2. Related Works

Many harsh environments have strong demands for localization, so there have been many related studies in recent years. RF-based indoor localization technology mainly relies on two features: time and RSS. There are also hybrid localization systems that combine with other sensors.

### 2.1. RSS Feature in Harsh Environments

The RSS feature is widely used in indoor localization technology and wireless sensor networks, but the reliability of RSS is doubtful in terms of improving the location accuracy [3]. The main reason why RSS is widely used is its simplicity and low cost. In fact, receivers (or beacon nodes) can easily read RSS in any wireless communication system. Therefore, in various wireless communications, such as Bluetooth, WiFi, RFID, and Zigbee, and RSS-based applications can be seen.

Compared with other RSS-based localization algorithms, the “fingerprinting” algorithm has a higher accuracy. In general, such algorithms are divided into two phases: the training phase and the implementation phase. In the training phase, a priori knowledge of the RSS distribution is received by the beacon node when a tag is in various locations in the environment (or map). Then, in the implementation phase, the real-time RSS of the tag, in every beacon node, will match with the map “fingerprinting”, and the location with the highest matching degree is the localization result. This solution has a high localization accuracy. Moreover, if the environment is stable, it still performs well in harsh environments [4,5,6]. However, it has two main drawbacks: (1) It requires a large amount of a priori data, and (2) the localization environment cannot be changed. Many studies have tried to overcome these shortcomings, for instance, [7] constructs a radio map with motion sensors, which makes it less demanding in terms of the priori data. However, the solutions presented have been unable to adapt to complex and changeable environments. 

Another RSS-based localization solution is to use the log-normal distance path loss (LDPL) model. The LDPL model describes the relationship between distance and RSS [8], and the trilateration algorithm can then be used to calculate the tag location. This solution does not require plenty of priori data like “fingerprinting”, and it can be more flexible in implementation. This has led to a great deal of related research. “iBeacon” is a popular commercial indoor localization technology [9], but its high accuracy is achieved by dense iBeacon beacon nodes. Therefore, it is not suitable for harsh environments. Hartman F proposes a fusion algorithm in article [10], which combines the IMU-based inertial navigation algorithm with RSS to achieve high-precision localization. However, the accuracy of the micro-IMU sensor is insufficient, and the inertial navigation algorithm is difficult to implement. However, it also requires a dense beacon node. Moreover, it is difficult to further improve. In short, the advantage of RSS is its low cost, not its localization accuracy. The RSS can be easily interfered with by many environmental factors, such as multipath, NLoS propagation, etc., which causes the relationship between distance and RSS to become extremely unreliable [3]. 

Obtaining RSSI or CSI information don’t require any additional operations or data on transmitters, which means that transmitters don’t consume extra energy for localization algorithms. This feature is also applied to other fields [11,12]. In addition, there is a channel state information (CSI) feature that can be used for indoor localization technology. It can be seen as an upgraded version of RSS. The CSI feature works in the physical layer. It can distinguish multipath propagation and weak fluctuation to a certain extent, thus depicting the energy characteristics of the signal. It performs better than RSS in most of cases. However, CSI has higher hardware requirements, and this information is also not sufficient to support high-precision localization in harsh environments [13].

### 2.2. Time Feature in Harsh Environments

Generally, time-based indoor feature technology can obtain a higher localization accuracy than RSS features. Common algorithms include time-of-flight (TOF)/time-of-arrival (TOA) and time difference of arrival (TDOA). In many cases, they can all achieve a centimeter-level localization accuracy, and this accuracy can meet the needs of most applications. 

The most popular communication solution for time-based indoor localization systems is ultra-wide band (UWB). The main reason for this is that UWB offers a realistic way of solving multipath problems and recording the propagation time [14]. However, its main drawback is that NLoS propagation caused by obstacles represents very serious interference, which will reduce the localization accuracy. The reason for this is that the frequency of UWB is very high, usually exceeding 3 GHz, while the penetration of high-frequency signals is weak. We can increase the power of UWB signals by using an amplifier to penetrate some obstacles (but the penetration is still not satisfied). However, multipath phenomena become a major source of error in TOA or TDOA estimation. This is because overlap of the arriving multipath is a source of error in the estimation of the first-arriving signal [15,16]. Moreover, some UWB signals cannot penetrate the obstacle, and this situation still exists in harsh environments. The solution to the NLoS problem mainly depends on identification and mitigation. For instance, Stefano Maran [17] evaluated the resulting performance based on various UWB propagation features through Monte Carlo simulations, and proposed an algorithm for identifying NLOS conditions and reducing the ranging error caused by NLOS conditions. The authors in articles [18,19] attempted to use the channel impulse response (CIR) from the antennas, together with ground truth locational data (derived by a robot equipped with an optical reference system), to train a deep convolutional neural network (CNN). Then, they used the CNN model to identify and mitigate the NLoS propagation. Indoor propagation channels can be divided into multiple categories so that the channel identification results can be used to evaluate how serious the NLOS effect is [20]. UWB signal attenuation is very serious in the case of NLoS propagation, and the time delay caused by NLoS will cause a large error in high-precision localization, both of which can be features for identifying and mitigating the NLoS [21,22,23]. 

However, all the solutions above for NLoS propagation in harsh environments have two drawbacks. First, when a tag transmits messages to beacon nodes, NLoS may occur simultaneously on multiple communication lines. These solutions have limits when facing this situation. Second, a special case of NLoS is that the signal does not reach the beacon node at all (i.e., it does not penetrate the obstacle). These solutions can hardly handle this situation. Unfortunately, these situations are common in harsh environments.

### 2.3. Hybrid System

All IMUs that can be embedded in tags are microelectric mechanical systems (MEMS). Currently, the accuracy of this kind of IMU cannot support strap-down inertial navigation systems for pedestrians. Although some works have tried to overcome the problem using UWB [24], we do not consider the inertial navigation-based algorithm. The IMU-based PDR algorithm is more common in pedestrian localization systems. The algorithm achieves localization by calculating the step size, the number of steps, and the direction of a pedestrian. This makes the PDR more resistant to external interference like NLoS propagation. However, there are cumulative errors over time. On the other hand, the UWB-based localization system has a high-accuracy, but can be seriously interfered with by NLoS propagation, multipath, etc. Accordingly, it is a good solution to combine both of the two technologies. 

There are a few works in the literature on combining UWB and PDR [25,26]. Tian proposed a low-cost inertial navigation system (INS) and UWB fusion pedestrian tracking system [26]. However, the system had only one UWB beacon node, and only UWB was used to correct the cumulative error of PDR. In Reference [27], the core idea is mainly to correct the PDR direction, but the parameters cannot be adjusted for each pedestrian. Qian proposes a fusion method for WiFi/magnetic matching/PDR in Reference [28], but the “fingerprinting”-based algorithm limits its application for harsh environments. Most of these hybrid systems have three drawbacks. Firstly, the calculation and storage resources of tags are limited, and complex algorithms cannot be deployed; secondly, everyone has their own walking characteristics, and these systems fail to dynamically adjust the walking parameters of each pedestrian; third, the fusion algorithm does not try to identify and mitigate the NLoS in UWB transmission. There is not much research related to this kind of solution. 

## 3. Problem Statement and System Architecture

In this section, we describe the problem in harsh environments for UWB-based and PDR algorithms. Then, we briefly introduce the sources of error of the two algorithms and the proposed hybrid indoor localization system architecture.

### 3.1. Environments and System Architecture

**Figure 1:**
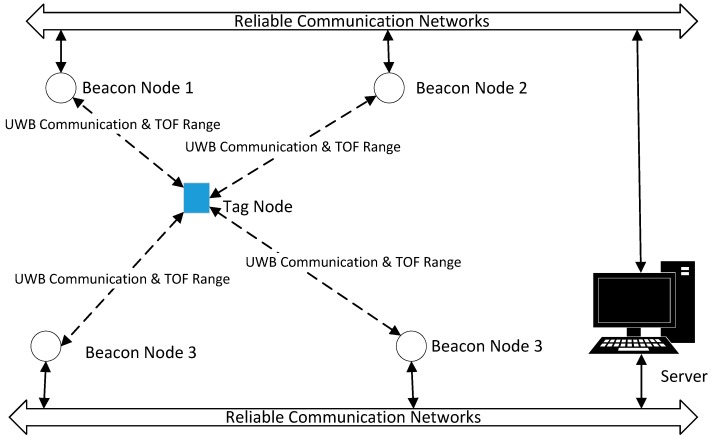
Communication structure of the radio frequency (RF)-based positioning system.

In this paper, the hybrid indoor localization system consists of three types of devices (or nodes): the tag node (TN), beacon node (BN), and server. The coordinates of BN are known. TN is a target to be located in the localization system. The pedestrian wears a TN in a fixed location on the body, such as his/her arm, hat, trouser, or jacket’s pocket. Each TN is an electric tag. The TN will embed a nine-axis inertial sensor: three-axis inertial sensor, three-axis gyroscope, and three-axis magnetometer. TN communicates with BN through UWB at a frequency of 1 Hz, which means that the UWB-based localization algorithm is executed once per second. The PDR algorithm will be implemented on TN. TN will upload the location results and download correction information by UWB communication. Meanwhile, TOF-based ranging is completed using three messages: the TN initiates the communication (sends the first message); the BN responds to the message (the second message); and finally, the TN replies with the final message (the final message). All message transmission and reception times will be recorded, and the TOF ranging algorithm can then be realized. BNs send all information to the server for final processing. The communication between the BN and server is considered reliable communication. In other words, this reliable communication will be considered to be real-time and not lose any packets in this paper. The whole communication structure can be seen in Figure 1.

In this paper, the localization algorithm is executed independently for every TN. Therefore, for notational convenience, we consider the point of view of one single TN in the system: The TN with unknown coordinate *p*, and surrounded by BNs with known coordinates *p_i_*, where *i* means the *i-*th BN.

### 3.2. Sources of Error and Problem Statement

There are harsh environments for the localization system. As a result, there will be interference in the environments, which can be sources of localization error. We will discuss them in the UWB-based and PDR algorithms separately. 

First, for the UWB-based localization algorithm, estimated distances have errors with respect to the true distances. In ideal LoS propagation, the ranging error is a Gaussian distribution because of the accuracy of hardware. In NLOS propagation, there are two cases: (1) Signals fail to penetrate obstacles, which we will mainly study in this article, and (2) signals fade with delay and/or attenuation. With the time-based algorithm, there will be a bias in ranging in this situation. Some other articles [17,20,23] have studied this situation and proposed approaches to identify and mitigate the bias. In this paper, we use the algorithm in [20] to mitigate the bias, and the Kalman filter is used to smooth the location results. Then, the accuracy of the results in this case can satisfy the demand of the hybrid system. 

Second, for the PDR algorithm, the erroneous results are not caused by external factors. Instead, the IMU sensor errors arise from random zero bias and oscillation noise. This is a cumulative error that cannot be self-eliminating.

In a hybrid system, the two localization algorithms will be realized separately in the first step. For the UWB-based algorithm, with a set of distance estimates calculated by the ToF algorithm, the TN’s location can be obtained using the least squares method (LSM). The biggest advantage of LSM is that it can compensate for the ranging errors to a certain extent. Its core idea is illustrated in Equation (1). Here, the TN’s coordinate, denoted as $p^{U}$, can be inferred by minimizing the cost function, and $p^{U}$ is equal to *p,* which minimizes *L*:

(1) $$L=\sum d_{i}-p-p_{i},\;p^{U}=arg\underset{p}{\min}L.$$

The PDR algorithm, based on the IMU embedded in the TN, estimates a pedestrian’s location, mainly depending on three algorithms: Step detection, stride length estimation, and heading determination. The coordinate of the pedestrian (TN) from PDR, denoted as $p^{D}\left(x_{t+1}^{D},y_{t+1}^{D}\right)$, can be iterated through the following equations:

(2) $$\{\begin{array}{c} x_{t+1}^{D}=x_{t}^{D}+LS*\sin\left(\theta \right)\\ y_{t+1}^{D}=y_{t}^{D}+LS*\sin\left(\theta \right) \end{array},$$

where *x* and *y* are the coordinates of the TN in a 2D map, *LS* is the stride length, and θ is the heading at epoch *t*. From Equation (2), it is shown that we can estimate the location of the pedestrian in real time when the initial location is known. 

The final location will be fused by a weighted average algorithm using the results of the two algorithms:

(3) $$P=w_{u}*P_{u}+w_{p}*P_{pdr},$$

where *P* is the final result, $P_{u}$ is the result from UWB, and $P_{pdr}$ is the result from the PDR algorithm. $w_{u}$ and $w_{p}$ are their weights, respectively, and can be calculated by experience.

## 4. Localization Using UWB

### 4.1. Ranging Algorithm Using ToF

The ToF ranging algorithm can be divided into two categories: Single-sided two-way ranging (SS-TWR) and double-sided two-way ranging (DS-TWR). SS-TWR uses one single round trip time measurement to obtain a time-of-flight result. Additionally, DS-TWR, an extension of the basic SS-TWR, uses two round trip time measurements, which results in a reduced error. Considering the communication structure and accuracy requirements in indoor localization systems, this paper chooses the DS-TWR strategy using three messages. Moreover, the three messages will provide three RSS to assist in calculating the weight $w_{u}$.

**Figure 2:**
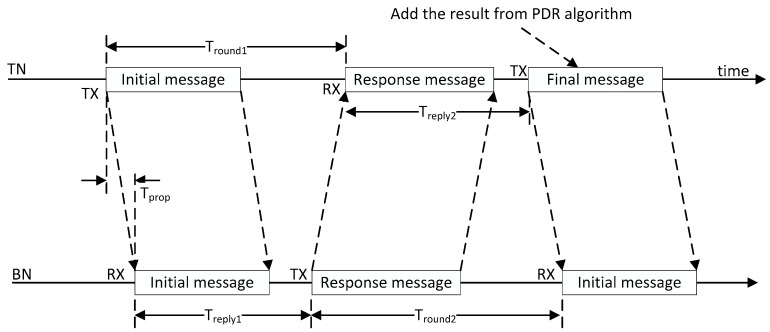
Three messages of double-sided two-way ranging (DS-TWR).

The three messages of DS-TWR are shown in Figure 2. The TN starts the initial message, and BNs receive it and respond with a second message. The TN receives the second message and transmits the final message. According to Figure 2, the time of signal flight $T_{prop}$ can be calculated, and the distance *d* will be the result of multiplying $T_{prop}$ by the speed of light *c*, shown as Equation (4):

(4) $$T_{prop}=\frac{T_{round1}*T_{round2}-T_{reply1}*T_{reply2}}{T_{round1}+T_{round2}+T_{reply1}+T_{reply2}},\;d=T_{prop}*c,$$

where $T_{round1}$ is the time interval between Initial message and Response message, and $T_{reply2}$ is the time interval between the response message and the final message in TN. $T_{reply1}$ is the time interval between the initial message and response message, and $T_{round2}$ is the time interval between the response message and the final message in BN.

### 4.2. Localization Algorithm

**Figure 3:**
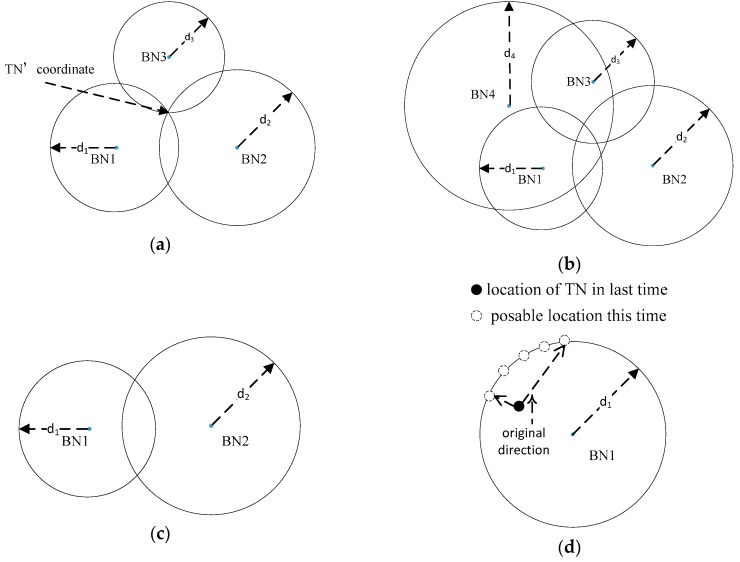
Tag node (TN) localization with different situations: (**a**) An ideal model with three beacon nodes (BNs); (**b**) real situation with ranging error; (**c**) localization with two BNs; (**d**) only one single BN completes the ranging step.

In an ideal model, with three known ranges between the TN and each of the three BNs, the coordinate of the TN can be obtained by trilateration. There are three circles, with the BN as the center and the distance as the radius. They are exactly intersecting at one point, based on Pythagorean theorem, which is the coordinate of the TN. This is illustrated in Figure 3a. However, the real situation is more complicated. Ranging errors mean that the three circles cannot intersect at one point. Moreover, the number of BNs may be more than three, as shown in Figure 3b. 

For real situations like Figure 3b, with ranging and coordinates of the BN, we can build the following equations:

(5) $${\left(x_{i}-x\right)}^{2}+{\left(y_{i}-y\right)}^{2}=d_{i}^{2},\;\;i=1,2,3,\ldots ,n,$$

where (*x, y*) is the coordinate of the TN, (*x_i_, y_i_*) means the *i-th* BN, and *d_i_* is the distance between the TN and the *i-*th BN. Based on these equations, we can employ the former (*n* − -1)-th equations minus the *n-*th. Then, we have (*n* − 1) equations. They are all linear functions and can be described in matrix form:

(6) $$\left[\begin{array}{cc} 2\left(x_{1}-x_{n}\right) & 2\left(y_{1}-y_{n}\right)\\ 2\left(x_{2}-x_{n}\right) & 2\left(y_{2}-y_{n}\right)\\ ... & ...\\ 2\left(x_{n-1}-x_{n}\right) & 2\left(y_{n-1}-y_{n}\right) \end{array}\right]\left[\begin{array}{c} x\\ y \end{array}\right]=[\begin{array}{c} x_{1}^{2}-x_{n}^{2}+y_{1}^{2}-y_{n}^{2}+d_{1}^{2}-d_{n}^{2}\\ x_{2}^{2}-x_{n}^{2}+y_{2}^{2}-y_{n}^{2}+d_{2}^{2}-d_{n}^{2}\\ ...\\ x_{n-1}^{2}-x_{n}^{2}+y_{n-1}^{2}-y_{n}^{2}+d_{n-1}^{2}-d_{n}^{2} \end{array}].$$

We can summarize Equation (6) as:

(7) Ax=b,

where $A=\left[\begin{array}{cc} 2\left(x_{1}-x_{n}\right) & 2\left(y_{1}-y_{n}\right)\\ 2\left(x_{2}-x_{n}\right) & 2\left(y_{2}-y_{n}\right)\\ ... & ...\\ 2\left(x_{n-1}-x_{n}\right) & 2\left(y_{n-1}-y_{n}\right) \end{array}\right]$, $X=\left[\begin{array}{c} x\\ y \end{array}\right]$, $b=\left[\begin{array}{c} x_{1}^{2}-x_{n}^{2}+y_{1}^{2}-y_{n}^{2}+d_{1}^{2}-d_{n}^{2}\\ x_{2}^{2}-x_{n}^{2}+y_{2}^{2}-y_{n}^{2}+d_{2}^{2}-d_{n}^{2}\\ ...\\ x_{n-1}^{2}-x_{n}^{2}+y_{n-1}^{2}-y_{n}^{2}+d_{n-1}^{2}-d_{n}^{2} \end{array}\right]$.

Based on the value of *A* in Equation (7), there are three cases to solve *X*.

**Case one**, *A = [0, 0]*. This means that only one single BN completes the ranging algorithm with TN, illustrated in Figure 3d. We cannot solve the exact coordinate of TN and can only get a possible range, even in an ideal model. Considering the motion model of TN in Figure 3d, we are able to narrow down the possible range. However, it is pointless in this localization system. There are two reasons for this: (1) ranging errors make the possible range larger and the (2) PDR algorithm has a higher accuracy in this situation. 

**Case two**, *A* is a singular matrix. This means that two BNs complete the ranging or all BNs are in a straight line. In this situation, we find the two BNs closest to the TN, as shown in Figure 3c. Then, we employ a binary quadratic equation:

(8) $$\{\begin{array}{c} {\left(x_{i}-x\right)}^{2}+{\left(y_{i}-y\right)}^{2}=d_{i}^{2}\\ {\left(x_{j}-x\right)}^{2}+{\left(y_{j}-y\right)}^{2}=d_{j}^{2} \end{array}.$$

To solve it, we will obtain two results. If we have the coordinate of the TN from the last step, we can then calculate the Euclidean distances between the coordinate and the two results. We choose the coordinate with a shorter distance as the final result this time.

**Case three**, *A* is not a singular matrix. This is a common situation in most localization research. In this situation, the number of BNs will be greater than or equal to three, and they are not in a straight line. According to Figure 3b, in order to improve the location accuracy, we use the LSM algorithm to calculate the coordinate instead of trilateration. Then, Equation (7) can be transformed to Equation (9). In this case, most UWB indoor localization systems can achieve a centimeter-level location accuracy.

(9) $$X={\left(A^{T}A\right)}^{-1}A^{T}b,$$

where *X* is the coordinate of the TN.

## 5. Localization Using PDR

The PDR algorithm is an IMU-based algorithm. Inertial navigation is an intuitive solution, but it is not suitable for tags for two reasons: (1) In a tag, limited by size and cost, the IMU is a microelectro mechanical system (MEMS), which makes the precision of IMU low, and (2) the strap-down inertial navigation system is too complicated to realize it in the TN. PDR focuses on step detection, stride length calculation, and heading estimation of the pedestrian. According to Equation (3), with an initial location, we can estimate the location at any moment.

### 5.1. Initial Location

The initial location is the premise of starting the PDR algorithm, but PDR itself cannot provide it. Therefore, we can use the result of UWB localization to provide it. According to the localization situation, the process of initializing the location can be divided into two cases:

(1) With Equation (7), matrix *A* is not a singular matrix. Then, we can calculate the coordinate of TN as the initial location, and start the PDR algorithm; and
(2) In other cases, we do not initialize the location or start the PDR algorithm, until case (1).

### 5.2. Step Detection

Step detection is a basic technique of PDR. It is crucial, but not complicated. There are many mature and efficient solutions. In this paper, we use the step detection algorithm from the [28].

The data for step detection comes from a triaxial accelerometer. Due to the difference in TN attitude, it is complicated to analyze the acceleration characteristics in each three-axis. The authors in [28] proposed a method using the total acceleration magnitude. The magnitude $a_{mag,t}$ at time *t* can be expressed as:

(10) $$a_{mag,t}=\sqrt{a_{x,t}^{2}+a_{y,t}^{2}+a_{z,t}^{2}},$$

where $a_{x,t}$, $a_{y,t}$, and $a_{z,t}$ are the measurements from the triaxial accelerometer at time *t*. Compared to the vertical direction of the acceleration, the total acceleration magnitude is insensitive to the orientation of a tag worn by pedestrians. Then, it can be adapted to different walking patterns. 

According to the value of $a_{mag}$, a candidate step can be detected by a threshold $\delta_{th}$, where the $a_{mag}$ has to cross $\delta_{th}$ from negative to positive. In order to ensure the accuracy of step detection, there are some constraints: (1) The time interval Δt between two consecutive steps must be within the interval threshold from $\Delta t_{min}$ to $\Delta t_{max}$; (2) the difference $a_{mag}$ between extreme values of $a_{mag}$ during a step phase and the threshold $\delta_{th}$ has to be between $a_{min}$ and $a_{max}$, otherwise a perturbation point is recorded; and (3) the threshold $\delta_{th}$ is updated dynamically according to the mean value of $a_{mag}$ over a step period, illustrated as:

(11) $$\delta_{th}=\frac{1}{m}\sum_{i=1}^{m}a_{mag}^{\left(i\right)},$$

where *m* means the number of samples of the accelerometer during a single step at the last point. 

The algorithm mentioned above can effectively detect steps when pedestrians walk or run, but it cannot work if pedestrians are standing. Different from [29], this paper uses another mature algorithm. When we detect a new candidate step, we record the two values—the maximum and minimum of $a_{mag}$—during the step. Only when the difference between the two values is greater than a threshold can, we consider the candidate step as a true step. The threshold can be obtained from a set of trained data. The data is a series of $a_{mag}$ when the pedestrians are walking or standing. We do not need to tag them, and we can use the K-means clustering algorithm to obtain the characteristics of $a_{mag}$ during different pedestrians’ motion. In K-means clustering algorithm, we consider Euclidean distance as the degree of difference between $\delta_{th}$, and cluster $\delta_{th}$ into two categories. The value of $\delta_{th}$ in the clustering center is smaller, which indicates a standing state, and larger, which indicates a walking state.

### 5.3. Stride Length Estimation

Stride length, combined with step detection, is utilized for the moving distance calculation of pedestrians. According to related PDR algorithm works, the stride length of a pedestrian is not constant and varies with walking speed, step frequency, acceleration variance, and so on. The relationship between these variables is linear, shown as:

(12) LS=α∗f+β∗v+γ,

where *LS* is the stride length; *f* is the step frequency; *v* is the acceleration variance; α and β are weights for step frequency and acceleration variance, respectively; and γ is the bias. In this paper, these three parameters are named walking parameters. In every step, v can be described as:

(13) $$v=\sqrt[{4}]{a_{max}+a_{min}},$$

where $a_{max}$ and $a_{min}$ represent the maximum and minimum of $a_{mag}$ in every single step, respectively.

In traditional algorithms, these parameters, including α, β, and γ, are measured by a set of offline data. This method will increase the deployment difficulty of the PDR algorithm. Moreover, every pedestrian has their own unique walking habit, which will lead to different walking characteristics, and will then lead to different parameters for each pedestrian. It is impossible to measure a set of parameters for each pedestrian. The hybrid system can solve the problem using results from UWB localization. 

The hybrid system can adaptively adjust walking parameters for each pedestrian. The core idea is that the precise location and walking trajectory obtained from the UWB-based localization algorithm will help the PDR algorithm to calculate the parameters. The whole process can be divided into two parts: the extraction of training data and linear regression for parameters.

The first step is training data extraction. Not all location results are suitable for training data. Therefore, we define a time window for a series of location points, and design a strategy to determine whether these points in the time window are suitable as training data. The length *l* of the time window is related to the frequency of localization. When the locations in the time window meet all the following three constraints, these locations in the window can be extracted as training data. 

(1) High precision of localization results

Training data with large errors will lead to parameters with errors. Therefore, we need to obtain an accurate location from the UWB-based algorithm. According to the description of the localization algorithm using UWB in Section 4, a centimeter-level location accuracy can be obtained in the case of non-singular matrix *A*. This accuracy can meet the needs of training parameters. As a result, we demand that all locations in the time window are calculated in the case of the non-singular matrix *A*.

(2) The pedestrian walks in a straight line

**Figure 4:**
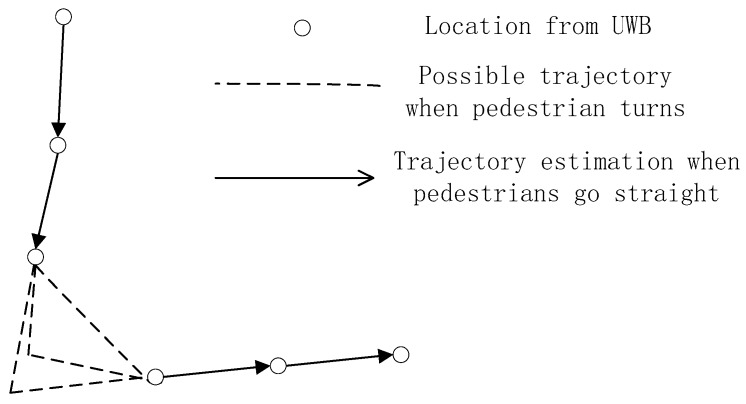
Trajectory estimation from Ultra-Wide Band (UWB)-based positioning results.

The frequency of the localization algorithm using UWB is low, because frequent localization will shorten the battery life of the TN. In this case, the accuracy of the walking trajectory estimation will be reduced when the pedestrian turns, as shown in Figure 4. Therefore, the locations in training data must be a straight line for pedestrians. The strategy for selecting location data will be described in Section 5.4.

(3) Pedestrian walks at a constant speed

A pedestrian’s walking speed is related to their stride length. Therefore, if a pedestrian walks at a different speed within a set of training data, their stride length is not constant. We can calculate the length separately with step detection, but there will be errors. With these cases, it is better to demand that the pedestrian walks at a constant speed when collecting training data. Fortunately, pedestrians walk at a constant speed most of the time. The strategy of selecting location data for walking with a constant speed will be described in Section 5.4.

Second, using these training data, the pedestrian trajectory can be obtained. With step detection, we are able to estimate the stride length in the time window. By repeating the first step, we can obtain multiple sets of training data and stride length. Using these data, we can perform a linear regression of Equation (12) to acquire the parameters.

### 5.4. Strategy for Selecting Location Data

**Figure 5:**
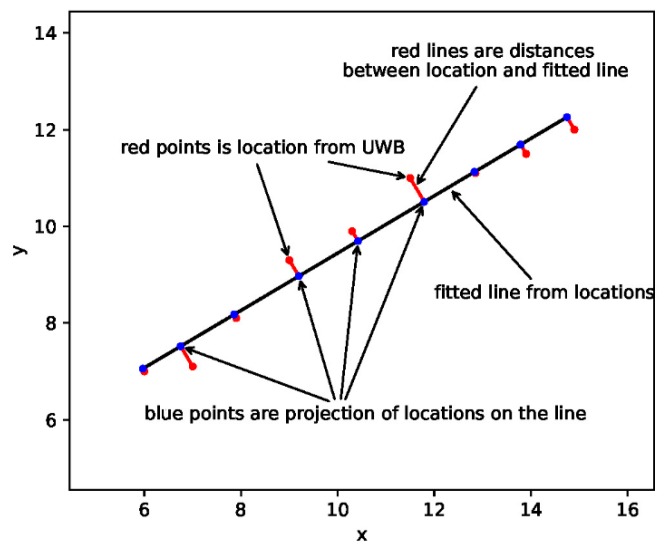
Trajectory estimation from UWB-based positioning results.

The location data from the UWB localization algorithm will be a series of discrete coordinates $\left(x_{i},\;y_{i}\right)$ on a localization map, as illustrated in Figure 5. They must be accurate according to the first constraint in Section 5.3. Figure 5 can be considered a small part of the location map. We can fit a line based on the coordinates. Then, the degree of dispersion of these points with the fitted line is used to determine whether the pedestrian walks in a straight line, and they are marked as red segments in Figure 5. The fitted line can be described as follows:

(14) y=ax+b.

Then, the process of fitting the line is employed to solve *a* and *b*. Using the coordinates, we have a series of equations:

(15) $$y_{i}=ax_{i}+b,\;i=1,2,3,\ldots ,n,$$

where $x_{i},y_{i}$ are the coordinates of location data. These equations can be expressed in the form of linear algebra as:

(16) $$Y=XB=>B={\left(X^{T}X\right)}^{-1}X^{T}T,$$

where $Y=\left[\begin{array}{c} y_{1}\\ y_{2}\\ ...\\ y_{n} \end{array}\right],X=\left[\begin{array}{cc} x_{1} & 1\\ x_{2} & 1\\ ... & ...\\ x_{n} & 1 \end{array}\right],B=\left[\begin{array}{c} a\\ b \end{array}\right]$.

We can calculate *a* and *b* through Equation (16), and then obtain the fitted line. The degree of dispersion can be described as the distance from each point to the fitted line:

(17) $$\xi =\frac{1}{n}\sum_{i=0}^{n}\left|\frac{ax_{i}-y_{i}+b}{\sqrt{a^{2}+1}}\right|.$$

Then, we can set a threshold $\xi_{th}$. In the time window, if $\xi <\xi_{th}$, we consider that the pedestrian walks in a straight line; otherwise, the pedestrian is not walking straight.

As shown in Figure 5, each location point has a projection on the fitted line, marked as blue points. By calculating the distances between all adjacent projection points, we can use the standard deviation σ of these distances to determine whether pedestrian walks at a constant speed. Then, we can still set a threshold $\sigma_{th}$, and if $\sigma <\sigma_{th}$, we can consider that the pedestrian walks at a constant speed; otherwise, the pedestrian is not walking at a constant speed.

The fitted line starts at the first projection and ends at the final projection. The length of this line is the distance the pedestrian walks. Considering it as a vector, the direction is the heading of the pedestrian, and will be used for heading estimation of the PDR algorithm in Section 5.5. It is the black line in Figure 5.

### 5.5. Heading Estimation

In this paper, the target of localization, the TN, is an electronic tag. Unlike a smartphone, the pedestrian wears the tag on a fixed location on their body. In this respect, the heading estimation in this PDR system is easier than the smartphone-based PDR algorithm. On the other hand, the basis of the heading estimation is the magnetometer, and the magnetometer can be disturbed in harsh environments. Therefore, drifting data from the magnetometer can cause heading prediction errors. We use three steps to solve this problem and estimate the heading direction.

Step 1:

We are able to calculate the yaw, pitch, and roll for the TN using the IMU-based attitude heading reference system (AHRS). In this paper, AHRS refers to the [30] and the algorithm details are not described here. Whenever new IMU data is obtained, the AHRS algorithm is executed once. According to Yaw, we have an angle $\theta_{Yaw}$ for the TN. However, this angle $\theta_{Yaw}$ refers to the direction of the electronic tag in the local Cartesian coordinates coordinate system (ENU). It is related to the attitude of the TN when the system is initialized and it has no connection with the heading of the pedestrian. Moreover, there will be an offset if we are trying to calculate the absolute direction in ENU using $\theta_{Yaw}$ and the magnetometer. 

**Figure 6:**
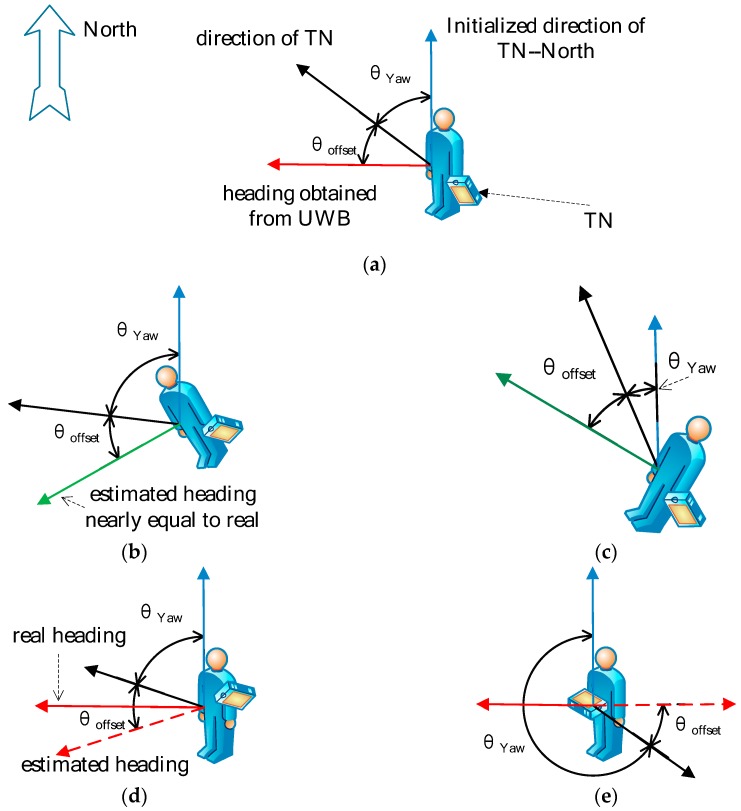
Heading estimation in different situations: (**a**) Obtaining the offset $\theta_{offset}$ between yaw and heading; (**b**,**c**) correct heading estimation; (**d**,**e**) wrong heading estimation.

Step 2:

Although the angle $\theta_{Yaw}$ has no connection with the heading, it still has a relationship with a certain absolute direction in ENU. Then, the problem is focused on finding a relationship between the directions with the heading of the pedestrian. According to Section 5.4, we can estimate the direction of the pedestrian using a set of location coordinates in a time window. Then, we have both the walking heading and $\theta_{Yaw}$ in the time window:

(18) $$\theta =\theta_{Yaw}+\theta_{offset},$$

where $\theta_{offset}$ is the offset between the heading and $\theta_{Yaw}$, as illustrated in Figure 6. As long as the pedestrian does not re-adjust the TN pose, it is constant for the TN in the location of the localization environment, as shown in Figure 6b,c, even if the magnetometer data exhibit errors caused by harsh environments.

Step 3:

Pedestrians may change the TN attitude in different walking states. Then, we can select the estimated heading when $a_{mag,t}=g$, where $a_{mag,t}$ is the magnitude $a_{mag,t}$ from the three-axis accelerometer at time *t* and g is the gravity acceleration. This represents the short period before the foot makes contact with the ground. If there is more than one $a_{mag,t}$, the average of the angles at $a_{mag,t}$ is taken as the result.

### 5.6. Location Estimation

With step detection, and stride and heading estimation, we are able to estimate the location during every step by Equation (2), as shown in Section 3.2. However, the PDR algorithm is applied in the TN, and the result must be transmitted to the server. UWB has its own protocol, so it is difficult to transmit the result in real time when the TN completes the algorithm.

Instead of estimating the location by Equation (2), we can combine the stride length and heading as a two-dimensional vector, and divide the vector by the time of the step. In this way, we can acquire the current pedestrian’s velocity vector. When the TN transmits a message to the BN, the TN estimates the current PDR location and adds it to the message.

## 6. Fusing the Two Results from UWB and PDR

With the location results from UWB and the PDR algorithm, the final result can be obtained by a weighted average using Equation (4) in Section 3.2. Then, the calculation of the weights can be concentrated on.

After UWB calibration, the localization accuracy of the PDR algorithm is very stable. UWB-based localization is vulnerable to the external harsh environment. If there is no NLoS in UWB communication, then the accuracy of UWB localization is higher than the PDR algorithm; otherwise, UWB is lower than PDR. Then, the weights depend on NLoS communication. 

According to the three descriptions of UWB localization in Section 4.2 and Equation (9), there are also three cases for weight determination and the decisive factor is also matrix *A*. 

First, *A =* [0, 0]. One single BN cannot provide location results by the UWB-based localization algorithm. Therefore, we do not use its result and make weights $w_{u}=0$ and $w_{p}=1$.

Second, A≠[0,0], but it is still a singular matrix. There are only two BNs completing ranging with the TN and they may also have NLoS transmission. In this case, the UWB localization system can complete the algorithm and provide a location result with errors. The accuracy of the results from the two localization algorithms becomes similar, which makes the weights similar. There are two ways of calculating the weight: (1) Through prior data, the accuracy of the two algorithms (represented by standard deviation) can be obtained, and the weights can then be determined by the ratio of the two standard deviation, or, (2) if it is difficult to obtain prior data, the weights can be set as $w_{u}=0.5$ and $w_{p}=0.5$. 

Third, *A* is not a singular matrix. In this case, the UWB localization system can provide an accurate location result that is more precise than PDR. We can set the weights as $w_{u}=0.9$ and $w_{p}=0.1$. Alternatively, standard deviation from prior data can be used to employ more precise weights.

## 7. Hybrid Localization System Implementation and Experiment

**Figure 7:**
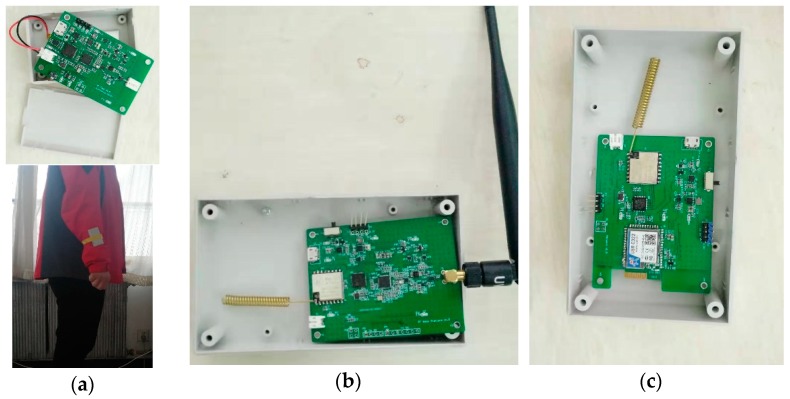
Three hardware devices for the hybrid localization system: (**a**) TN; (**b**) BN; (**c**) middleware.

In order to deploy our hybrid localization system, we have designed three hardware devices: TN, BN, and middleware. They are shown in Figure 7. The TN installs mpu9250 as the IMU, and uses dw1000 to make UWB communication and ranging with the BN. In order to make the UWB communication distance further, we installed power amplifiers on both TN and BN. Additionally, in order to facilitate the aggregation of data on the BNs, we have designed a middleware. It communicates with BNs by LoRa and realizes the convergence of location data, and then sends data to the server via WiFi. In these experiments, after employing power amplifiers, the UWB communication distance can reach at least 212 meters in the case of LoS. The theoretical communication distance of LoRa is more than five kilometers. The middleware uses polling to communicate with BNs, and the distance between them is less than 300 meters. The middleware communicates with the server via WiFi, and the distance between them is less than 5 m. We have tested the reliability of LoRa and WiFi in this environment. In total, 72,000 packets were transmitted in one hour, and the number of lost packets was 0. Therefore, we can consider LoRa and WiFi communication as reliable within this distance. 

The TN reads IMU data at a frequency of 20 Hz. Uploading these data to the server is a significant burden for UWB communication. There are two main reasons for this: (1) Transmitting a large amount of data will increase the power consumption of the TN, thereby shortening the battery life, and (2) these data demand a higher UWB bandwidth, but a high bandwidth will reduce the communication distance. As a result, we can run the PDR algorithm in the TN. When the TN and BN are ranging by UWB with three messages, the TN uploads the real-time location and downloads the speed and heading of the TN. All ranging data and location results from PDR will be sent to the middleware and then uploaded to the server by WiFi. The hybrid localization system needs some preset parameters, and their values are shown in Table 1.

**Figure 8:**
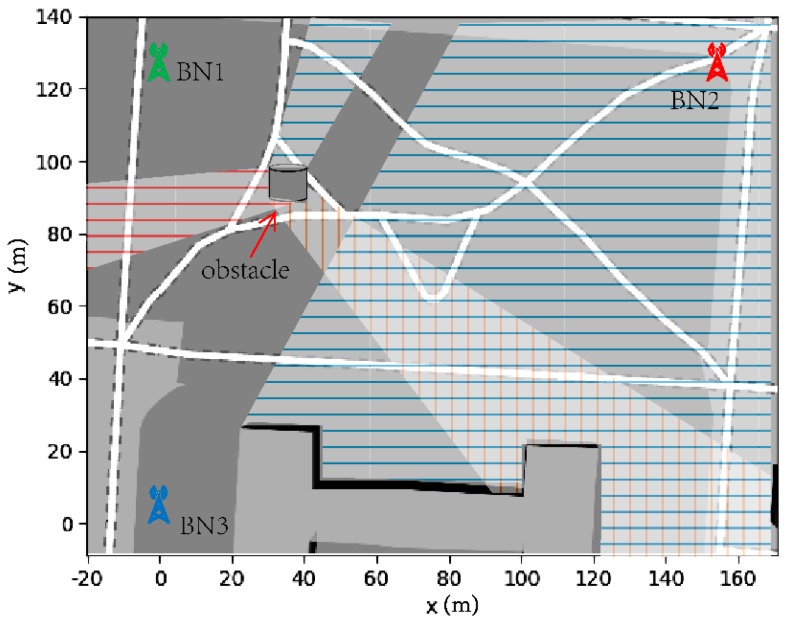
Deployment of BNs and UWB communication coverage situation.

The localization environment for the experiment was set as a small park in a university, as shown in Figure 8. We used three BNs and one TN, and the coordinates of the three BNs were known. Figure 8 shows their location and labels them with different colors. Due to some obstacles, including trees and a model, there are some areas in the park which mean that TN can only communicate with one or two BNs (NLoS communication). In Figure 8, we have used different color shades to indicate the loss of the BN signal. There are also large enough areas with non-NLoS communication, providing a high-precision location from the UWB-based localization algorithm to train parameters for the PDR algorithm. This situation can satisfy the experiment. 

With the platform as described above, we ran the hybrid localization algorithm. Additionally, we also implemented a UWB-based localization and PDR algorithm as a comparison. The real trajectory and coordinates calculated the errors of localization algorithms’ results. In order to ensure the accuracy of the real trajectory, the tester walked on a fixed trajectory with a fixed speed. We recorded the time when the tester passed through key reference objects, and then compared the distance and UWB high-precision localization results to obtain high-precision real trajectories and coordinates. We repeated the experiment 30 times. 

**Figure 9:**
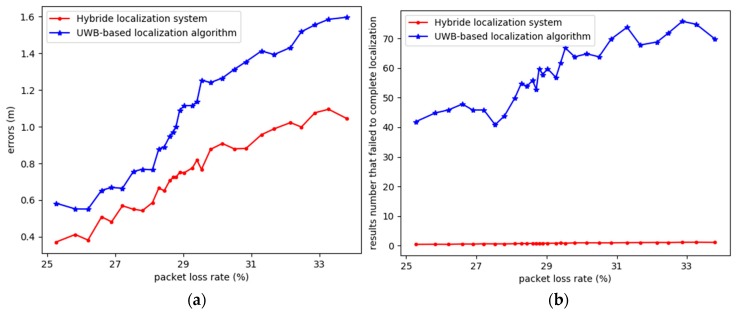
Comparison with different packet losses: (**a**) Average errors comparison vs. packet loss rate; (**b**) location results that failed to complete the localization algorithm vs. packet loss rate.

During the experiment, obstacles such as trees and models caused UWB communication packet loss, which means different accuracies for the algorithms. The comparison of results the UWB-based and hybrid localization system are illustrated in Figure 9. Figure 9a shows the average errors from the UWB-based and hybrid localization system, and only coordinates with successful completion from the localization calculation are counted. Since there are obstacles blocking UWB signals, the localization algorithm may fail to complete the calculation. Figure 9b shows the location results that failed to complete the calculation. We can see that the hybrid system is more robust to signal blocking. In other words, the hybrid system is more robust and accurate in harsh environments.

**Figure 10:**
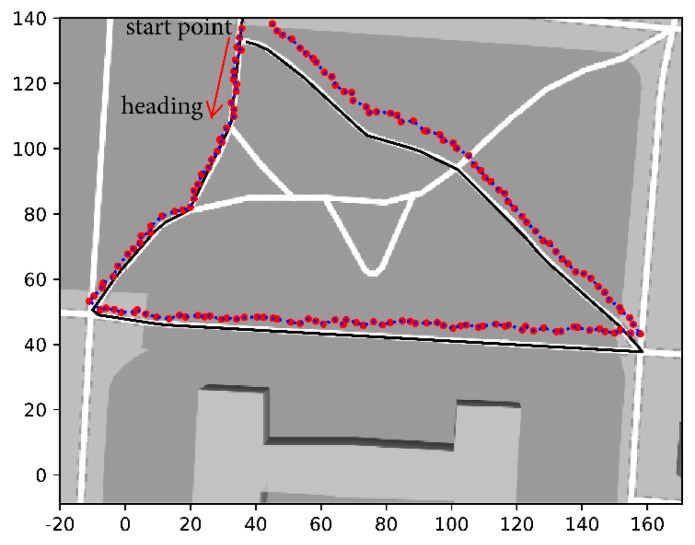
Location results from the pedestrian dead reckoning (PDR) algorithm.

**Figure 11:**
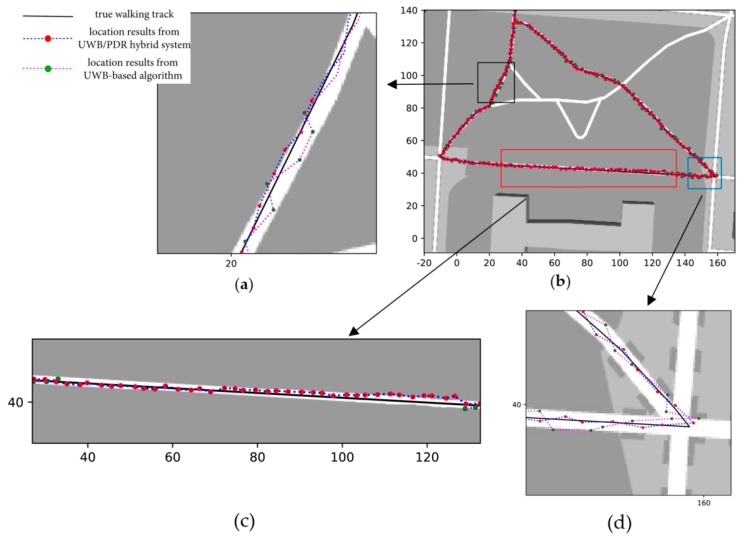
Comparison of experimental results from different localization algorithms: (**a**) Zoomed-in on the black frame in Figure b; (**b**) a comparison of the two localization results: UWB and hybrid system; (**c**) zoomed-in on the red frame in Figure b; (**d**) zoomed-in on the blue frame in Figure b.

Figure 10 and Figure 11 show a comparison of the experimental results obtained from different localization algorithms. Figure 10 shows the results from the PDR algorithm, where the black line is the true walking track and the red points are the location results acquired from the PDR algorithm. The PDR algorithm uses trained parameters from the UWB-based algorithm. We can see that as the walking distance increases, the offset of the locations becomes bigger. In Figure 11b, a comparison of the UWB-based algorithm and hybrid system is presented. The map is too big to differentiate the details of the two algorithms. Therefore, we zoomed in on parts of the map, as shown in Figure 11a,c,d, and marked them with different color frames in Figure 11b. The black arrows point to the zoomed-in figures. 

Referring to Figure 8, we can confirm that the TN can communicate with only two BNs in the areas with a black and blue frame in Figure 11b, which meets the second case described in Section 4.2. We zoomed-in on the results in Figure 11a,d. In the two figures, we can see that the location results obtained from the UWB-based algorithm have a higher dispersion than the UWB/PDR hybrid localization system. The mean of the results is similar to the true walking track, but the standard deviation of the UWB-based algorithm is larger. In other words, the errors of the UWB-based algorithm are larger. In case two, according to all the experimental results, the total average error of the UWB-based algorithm is 2.12 meters, and that of the hybrid system is 0.71 meters. Figure 11c shows the results in case one. In this situation, the TN can communicate with only one BN, and the UWB-based algorithm cannot complete the location calculation. In the figure, we can see that we cannot obtain the location from the UWB-based algorithm. We can still obtain location results from the PDR system, although the results begin to drift with an increasing distance. However, once the localization situation returns to case two or three, the results are quickly recalibrated. In this case, the offset error per 100 meters of the hybrid localization system is 1.3 meters.

The advantage of PDR in the hybrid system is that it can dynamically adjust the walking parameters for each pedestrian. This makes the algorithm more robust for different pedestrians. In the walking experiments, a male student with a height of 1.82 m completed the test 20 times, a female student with a height of 1.69 m completed the test 10 times, and a female student with a height of 1.55 m completed it 10 times. We saved the data in the flash of MCU, and use the same data to run a PDR algorithm in [28] with fixed parameters and the hybrid system. By manually adjusting the parameters for the male student a height of 1.82 m, the offset error per 100 m of PDR in [28] could achieve 1.18 m, which is better than the hybrid. However, if we used the parameters for the female student, the offset error per 100 m would be 2.1 m. The compressions between PDR in [28] and hybrid system are illustrated in Table 2 and Table 3.

## 8. Conclusions

In this paper, we have proposed a hybrid localization system for fusing the UWB-based and PDR algorithm. The UWB-based localization algorithm has a high accuracy and weak anti-interference ability. PDR is based on internal sensors and is less affected by external influences, but it produces cumulative errors that cannot be self-eliminating. We used accurate location results from the UWB-based algorithm to train parameters in PDR to improve its accuracy. Then, PDR was used to improve the localization accuracy when UWB was disturbed by external interference. The experimental results show that the accuracy of the hybrid system is higher than that of UWB or PDR. However, when the system is running, its greatest drawback is that the TN consumes more power, which will shorten the battery life. Future work needs to focus on low-power algorithms. Recently, deep learning has been proven to be a powerful tool, and has been successfully applied in many fields [31,32,33]. We will try to use deep learning algorithms to train the data in the future to obtain higher accuracy location results. Besides, TDOA based localization systems have greater capacity of tags than TOF-based. We will learn from some of new communication approaches [34,35,36] to achieve hybrid localization based on TDOA algorithm in the next works.

## Figures and Tables

**Table 1:** Preset parameters for the hybrid localization system.

Symbols	Parameters Description	Values
$\left(w_{u}^{1},w_{p}^{1}\right)$	Weights when *A =* [0, 0]	(0, 1)
$\left(w_{u}^{2},w_{p}^{2}\right)$	Weights when *A* is a singular matrix.	(0.5, 0.5)
$\left(w_{u}^{3},w_{p}^{3}\right)$	Weights when *A* is not a singular matrix.	(0.9, 0.1)
$\xi_{th}$	Threshold for judging whether to walk straight.	0.8
$\sigma_{th}$	Threshold for judging whether to walk at a constant speed.	0.8

**Table 2:** Errors with PDR in the hybrid system.

Pedestrians	Gender	Height	Average Errors per 100 m (m)	Max Error per 100 m (m)
a	male	1.82 m	1.3	1.51
b	female	1.69 m	1.33	1.56
c	female	1.55 m	1.28	1.49

**Table 3:** Errors with PDR in [28].

Pedestrians	Gender	Height	Average Errors per 100 m (m)	Max Error per 100 m (m)
a	male	1.82 m	1.18	1.46
b	female	1.69 m	2.3	2.80
c	female	1.55 m	2.1	2.75
